# Supplementary material for: Effect of Material and Process Variables on Characteristics of Nitridation-Induced Self-Formed Aluminum Matrix Composites—Part 1: Effect of Reinforcement Volume Fraction, Size, and Processing Temperatures
Source: Materials (Basel). 2020 Mar 13;13(6):1309. doi: 10.3390/ma13061309 (PMC7143127; doi:10.3390/ma13061309)
Supplement: Supplementary file 1 [file materials-13-01309-s001.pdf]

materials

SUPPLEMENTARY

Effect of Material and Process Variables on Characteristics of Nitridation-Induced Self-Formed Aluminum Matrix Composites—Part 1: Effect of Reinforcement Volume Fraction, Size, and Processing Temperatures

Dae-Young Kim, Pil-Ryung Cha, Ho-Seok Nam, Hyun-Joo Choi * and Kon-Bae Lee *

School of Advanced Materials Engineering, Kookmin University, 02707, Seoul, South Korea; kdy6603@kookmin.ac.kr (D.-Y.K.); cprdream@kookmin.ac.kr (P.-R.C.); hsnam@kookmin.ac.kr (H.-S.N.)

* Correspondence: hyunjoo@kookmin.ac.kr (H.-J.C.); kblee@kookmin.ac.kr (K.-B.L.); Tel.: +82-2-910-4287 (H.-J.C.); +82-2-910-4230 (K.-B.L.)

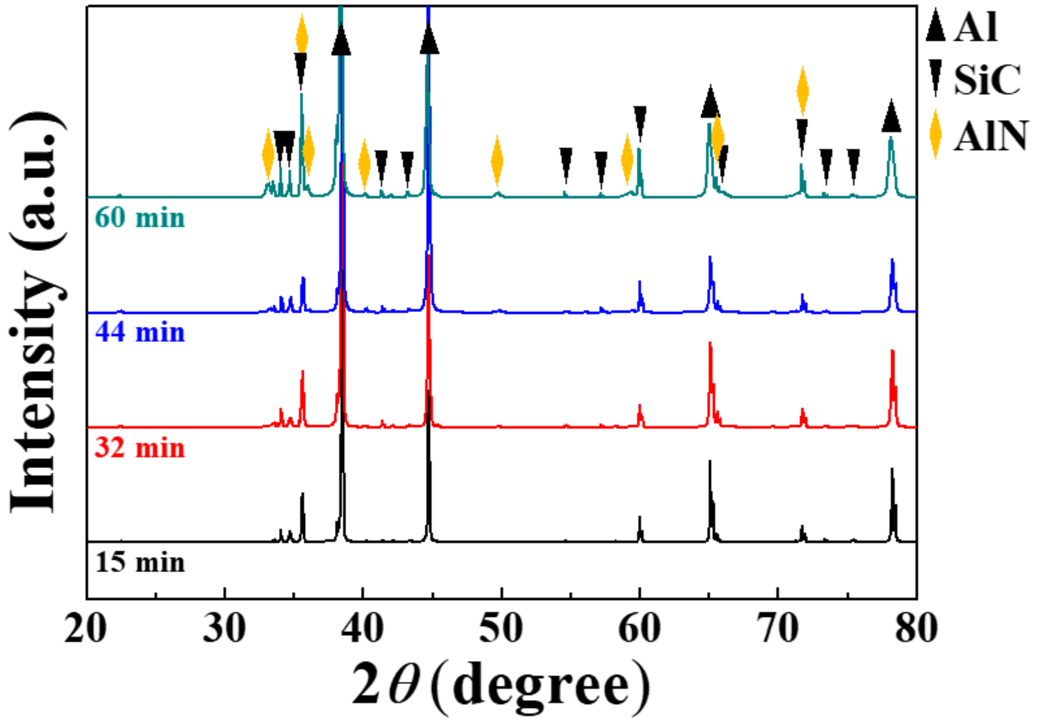

Figure S1. XRD patterns of the 20 vol.%SiC/6061Al powder beds held at 650 °C for various times.
